# Porin Involvement in Cephalosporin and Carbapenem Resistance of *Burkholderia pseudomallei*

**DOI:** 10.1371/journal.pone.0095918

**Published:** 2014-05-01

**Authors:** Anuwat Aunkham, Albert Schulte, Mathias Winterhalter, Wipa Suginta

**Affiliations:** 1 Biochemistry-Electrochemistry Research Unit, Schools of Chemistry and Biochemistry, Institute of Science, Suranaree University of Technology, Nakhon Ratchasima, Thailand; 2 School of Engineering and Science, Jacobs University, Bremen, Germany; University of Cambridge, United Kingdom

## Abstract

**Background:**

*Burkholderia pseudomallei* (*Bps*) is a Gram-negative bacterium that causes frequently lethal melioidosis, with a particularly high prevalence in the north and northeast of Thailand. *Bps* is highly resistant to many antimicrobial agents and this resistance may result from the low drug permeability of outer membrane proteins, known as porins.

**Principal Findings:**

Microbiological assays showed that the clinical *Bps* strain was resistant to most antimicrobial agents and sensitive only to ceftazidime and meropenem. An *E. coli* strain defective in most porins, but expressing *Bps*Omp38, exhibited considerably lower antimicrobial susceptibility than the control strain. In addition, mutation of Tyr119, the most prominent pore-lining residue in *Bps*Omp38, markedly altered membrane permeability, substitution with Ala (mutant *Bps*Omp38Y119A) enhanced uptake of the antimicrobial agents, while substitution with Phe (mutant *Bps*Omp38Y119F) inhibited uptake. Channel recordings of *Bps*Omp38 reconstituted in a planar black lipid membrane (BLM) suggested that the higher permeability of *Bps*Omp38Y119A was caused by widening of the pore interior through removal of the bulky side chain. In contrast, the lower permeability of *Bps*Omp38Y119F was caused by introduction of the hydrophobic side chain (Phe), increasing the ‘greasiness’ of the pore lumen. Significantly, liposome swelling assays showed no permeation through the *Bps*Omp38 channel by antimicrobial agents to which *Bps* is resistant (cefoxitin, cefepime, and doripenem). In contrast, high permeability to ceftazidime and meropenem was observed, these being agents to which *Bps* is sensitive.

**Conclusion/Significance:**

Our results, from both *in vivo* and *in vitro* studies, demonstrate that membrane permeability associated with *Bps*Omp38 expression correlates well with the antimicrobial susceptibility of the virulent bacterium *B. pseudomallei*, especially to carbapenems and cephalosporins. In addition, substitution of the residue Tyr119 affects the permeability of the *Bps*Omp38 channel to neutral sugars and antimicrobial agents.

## Introduction

*Burkholderia pseudomallei* (*Bps*) is a Gram-negative bacterium that causes melioidosis, a highly infectious disease that is endemic to areas that include south and southeast Asia and northern Australia [1]–[4]. Thailand has the highest recorded incidence of melioidosis in the world, especially in the north-eastern region, with an average 21.3 cases per 100,000 people per year [5]. Here, 80% of children are positive for antibodies against *Bps* by the age of 4 [6]. Patients infected with *Bps* usually develop skin ulcers, visceral abscesses, pneumonia and septicaemia and require urgent antimicrobial treatment to avoid fatal progression of the disease [7], [8]. However this is difficult, since *Bps* is highly resistant to most classes of antimicrobial agent. Melioidosis patients are usually treated with broad-spectrum antibiotics such as intravenous ceftazidime, meropenem, and imipenem. In more severe cases, a combination of cefoperazone and sulbactam may be administered, but the survival rate is relatively low at 60%, though a combination of ceftazidime and cotrimoxazole may help to increase this to 70–75% [9]–[12]. If these drugs are unavailable, amoxicillin-clavulanate (co-amoxiclav) may be used, but is often ineffective [13].

*Bps* is regarded a Tier 1 Select Agent [14] and has been listed by the US Centre for Disease Control and Prevention as a category B health hazard [15], [16]. The severe biosafety concerns associated with *Bps* have prompted biomedical research into drug resistance mechanisms during melioidosis infection, the ultimate goal being the development of effective anti-*Bps* agents. As with many multidrug-resistant pathogenic bacteria, the possible mechanisms underlying antimicrobial resistance of *Bps* are diverse, and include: i) that the porins in the outer membrane of *Bps* are of low permeability, thereby preventing the permeation of antimicrobial agents into the bacterial cytoplasm; ii) that *Bps* expresses effective efflux pumps that promptly export antimicrobial agents that have entered the cell; and iii) that *Bps* produces modifying enzymes that can deactivate or modify drug molecules [17]–[21].

We previously isolated an outer membrane protein with an apparent molecular weight of 38 kDa, referred to as *Bps*Omp38, from the cell wall of *Bps* [22], [23]. Topology prediction and molecular modelling suggested that *Bps*Omp38 has a *β*-barrel structure, a feature common among porins [23]. In a previous study, we cloned the gene encoding *Bp*sOmp38 into the pET23d(+) expression vector and expressed it in the Omp-deficient *E. coli* Omp8 Rosetta strain. The recombinant *Bps*Omp38 was reconstituted into black lipid membranes (BLM), and single channel recordings in the presence of seven antimicrobial agents demonstrated that the translocation rate through the *Bps*Omp38 channel depended on the molecular size and structure of the antimicrobial agent [24]. In the present study, we verified that heterologous expression of *Bps*Omp38 in Omp-deficient *E. coli* host cells lowers their antimicrobial susceptibility, compared to the control cells. We further employed site-directed mutagenesis, together with BLM measurements, to demonstrate the role of a prominent residue, Tyr119, protruding into the lumen of the *Bps*Omp38 pore. Electrophysiological results agree with both biochemical and microbiological assays, and suggest that Tyr119 exercises control of the permeation of ions, neutral sugars and certain antimicrobial agents through the *Bps*Omp38 channel.

## Materials and Methods

### Bacterial Strains and Vectors

Our clinically-derived strain was obtained from Thummasart University Hospital, Bangkok, Thailand. *E. coli* strain DH5*α,* used for routine cloning and plasmid preparations, was obtained from Invitrogen (Gibthai Company, Ltd. Bangkok, Thailand). *E. coli* BL21(DE3) Omp8 Rosetta mutant strain was a gift from Professor Dr. Roland Benz, Jacobs University Bremen, Germany. This *E. coli* strain, engineered to have defective genes encoding the major outer membrane porins OmpA, OmpC, OmpF and LamB [25], was suitable for production of the heterologous *Bps*Omp38. The pGEM-T easy vector, used for subcloning, was a product of Promega (Promega Pte Ltd, Singapore Science Park I, Singapore) and pET23d(+) expression vector was a product of Novagen (Merck Ltd., Bangkok, Thailand).

### Antimicrobial Susceptibility Assay

MIC values were determined from 4–6 trials (the reaction set up in triplicate for each trial) by the Mueller-Hinton (MH) two-fold dilution method, following the Clinical and Laboratory Standards Institute (CLSI) guidelines [26]. Different classes of antimicrobial agents used for susceptibility tests included: class 1 (penicillins): penicillin G and amoxicillin; class 2 (cephalosporins): ceftazidime, cefoxitin and cefepime; class 3 (carbapenems): meropenem, imipenem and doripenem; class 4 (fluoroquinolones): norfloxacin and ciprofoxacin; class 5 (quinolone carboxylic acid): enrofloxacin; class 6 (sulfonamide-trimethoprim): co-trimoxazole; and class 7 kanamycin and gentamicin. MIC values were evaluated with the cells grown in the presence of 50 µg.mL^−1^ phenylarginine-β-naphthylamide (PAβN), an inhibitor of RND-type multidrug efflux pumps [27], [28]. *B. pseudomallei* or *E. coli* BL21 (DE3) Omp8 Rosetta, transformed with an empty pET23d(+) vector or with the recombinant vector pET23d(+)/*BpsOmp38*, were streaked onto LB agar plates and incubated at 37° for 16 h. Single colonies of the bacteria were grown in 5 mL of MH medium and incubated at 37° for an additional 16 h. To assess the effect of the plasmid-derived *β*-lactamases on penicillin antimicrobial agents, 10 µL aliquots of the cell culture (diluted to OD_600_ of 0.1 or equivalent to 10^4^–10^5^ CFU^.^mL^−1^) were transferred into a 96-well plate, each well containing 90 µL LB medium and two-fold dilutions of penicillin G or amoxicillin mixed with clavulanic acid in a ratio of 2∶1. After incubation at 37° for 24 h, MIC values were evaluated in comparison with the breakpoints for *pseudomonas spp*. as recommended by the European Committee on Antimicrobial Susceptibility Testing-EUCAST (http://www.eucast.org/).

### Structure Prediction

The structural model of *Bps*Omp38 was built based upon the Modeller suite of programs as described by Suginta *et al.* [24]. The mutated structures of *Bps*Omp38Y119A and *Bps*Omp38Y119F were created using the WinCoot program (http://www.ysbl.york.ac.uk/~emsley/coot) and displayed with PyMol (v. Education-Use-Only) (www.pymol.org).

### Cloning and Site-directed Mutagenesis

Full-length *BpsOmp38* cDNA, including the *N*-terminal signal sequence, was inserted in the pET23d(+) vector so that the expressed *Bps*Omp38 could readily insert into the cell wall of *E. coli* BL21(DE3) host cells. The recombinant *Bp*sOmp38 was expressed as a hexahistidine-tagged protein so that it could be purified by affinity chromatography. For cloning, the *p*GEM-T/*BpsOmp38* construct from our laboratory was used as DNA template. The forward primer used for PCR amplification was 5′-TACCATGGCAAATAAGACTGATTGTTG-3′. This sequence included an *Nco*I restriction site followed by the initiation codon ATG and nucleotides that encode the start of the 20-amino acid *N*-terminal signal peptide of *Bps*Omp38. The reverse primer was 5′-TACTCGAGGAAACGTGACGCAGACC-3′. Gene amplification was carried out with Fermentas Pƒu DNA polymerase (Bio-Active Co., Ltd., Bangkok, Thailand) using a GeneAmp PCR System 9700 Thermocycler (Applied Biosystems, Foster City, CA, USA). The PCR product of the expected size (1.1 kb) was cloned into the plasmid pET23d(+), and then transformed into *E. coli* DH5α cells using a standard cloning protocol.

For site-directed mutagenesis, the newly generated construct pET23d(+)/*BpsOmp38* was used as DNA template in a PCR-based strategy. For *Bps*Omp38Y119A, the forward and reverse primers were respectively 5′-CTGGGCCGTCAGGCCGACGCAACCCAAGAC-3′ and 5′-GCTTTGGGTTGCGTCGGCCTGACGGCCCAG-3′. For *Bps*Omp38Y119F, the forward and reverse primers were respectively 5′-GGGCCGTCAGTTCGACGCAACCCAAG-3′ and 5′-CTTGGGTTGCGTCGAACTGACGGCCC-3′. The underlined sequences represent the mutated codons. Site-directed mutagenesis was performed following the QuikChange Site-Directed Mutagenesis protocol of Stratagene. The *Dpn*I-treated DNA was then transformed into *E. coli* XL1-Blue competent cells. The recombinant plasmids obtained from positive colonies were extracted using QuickClean II Plasmid Miniprep Kits (GenScript, Piscataway, NJ, USA). The recombinant plasmids, designated pET23d(+)/*BpsOmp38Y119A* and pET23d(+)/*BpsOmp38Y119F*, were re-transformed into *E. coli* DH5α cells. To verify that mutations were correct, the nucleotide sequences of the sense and anti-sense strands of the PCR fragment were determined by automated sequencing (First BASE Laboratories Sdn Bhn, Selangor Darul Ehsan, Malaysia).

### Protein Expression and Purification

Expression and purification of the recombinant *Bps*Omp38 variants were carried out as previously described [24]. In brief, transformed cells were grown at 37° in Luria-Bertani (LB) liquid medium containing 100 µg^.^mL^−1^ ampicillin. At an OD_600_ reading of 0.5, IPTG (isopropyl β-D-thiogalactoside) was added to a final concentration of 0.4 mM. Cell growth was continued for a further 6 h and then cells were harvested by centrifugation at 2,948 ×*g* for 10 min. The cell pellet was re-suspended in buffer containing 20 mM Tris-HCl, pH 8.0, 2.5 mM MgCl_2_, 0.1 mM CaCl_2_, 10 µg^.^mL^−1^ DNase I and 10 µg^.^mL^−1^ RNase A and then disrupted using a high-pressure homogenizer (EmulsiFlex-C3, Avestin Europe, Mannheim, Germany). The recombinant *Bps*Omp38 was further extracted from the peptidoglycan layer using sodium dodecyl sulfate (SDS)-containing solutions, based on a procedure reported by Lugtenberg and Alphen [29]. Briefly, SDS was added to the cell suspension to a final concentration of 2% (w/v) and incubation carried out for 1 h at 60° with gentle shaking. The crude extract was then centrifuged at 40,000 ×*g* for 60 min at 4°. The pellet, which at this stage included the cell envelopes, was re-suspended in 20 mM phosphate buffer (PBS), pH 7.4, containing 0.125% (v/v) octyl-POE (n-octyl-polyoxyethylene; ALEXIS Biochemicals, Lausanne, Switzerland). The suspension was incubated at 37° with gentle shaking for 60 min and then centrifuged at 100,000 ×*g* at 4° for 40 min. The new pellet, rich in outer membranes, was resuspended in 20 mM PBS, pH 7.4 containing 3% (v/v) octyl-POE and the suspension incubated at 37° with gentle shaking for 1h to solubilize the porin. Insoluble material was removed by centrifugation at 100,000×*g* at 20° for 40 min and the porin-rich supernatant concentrated and exchanged into a new buffer containing 0.2% (v/v) LDAO (lauryldimethylamine oxide; Sigma-Aldrich Pte. Ltd., Singapore), using Amicon Ultra-15 centrifugal filter devices with a nominal MW limit of 30 kDa (Millipore, Schwalbach, Germany). *Bps*Omp38 was further purified by cation exchange chromatography using a Hitrap SP HP column (5×1 mL) connected to an ÄKTA Prime plus FPLC system (GE Healthcare Life Sciences, Life Sciences Instruments, ITS (Thailand) Co., Ltd., Bangkok, Thailand). The chromatography was performed at 25° with a constant flow rate of 1.0 mL^.^min^−1^. *Bps*Omp38 was eluted with a linear gradient of 0–1 M KCl in 20 mM Tris-HCl, pH 8.0 containing 0.2% (v/v) LDAO. The purity of the eluted proteins was confirmed by SDS-PAGE. Fractions containing only *Bps*Omp38 were pooled and the protein concentration was determined using the Pierce BCA protein assay kit (Bio-Active Co., Ltd., Bangkok, Thailand).

### Immunological Analysis

Immunoblotting was performed following the standard ECL protocol. Purified *Bps*Omp38 (5 µg) was resolved on a 10% polyacrylamide-SDS gel, and after electrophoresis the protein was transferred to a nitrocellulose membrane using a Trans-Blot SD semi-dry electrophoretic transfer cell (Bio-Rad Laboratories Ltd., Bangkok, Thailand). Cross-reactivities of different porins were tested using specific antibodies against *Bps*Omp38, *E. coli* OmpF, *E. coli* OmpN, and *Vibrio harveyi Vh*ChiP. Signals representing antibody-antigen interaction were detected with HRP-conjugated IgG, using the enhanced chemiluminescence method (ECL, Amersham, UK). Rabbit anti-*Bps*Omp38 serum was prepared in our laboratory as described by Siritapetawee *et al*. [23].

### Electrophysiology

A black lipid membrane (BLM) reconstitution technique was used to demonstrate the pore-forming properties of the *Bps*Omp38 variants. Ion channel measurements were performed as described elsewhere [30]. Briefly, the BLM setup included a patch clamp amplifier with a two-electrode bilayer head-stage (PC-ONE plus PC-ONE-50, Dagan Corporation, Minneapolis, MN, USA), a Faraday cage placed on a vibration-dampening table, an A/D converter and software for computer-controlled operation (PULSE program, HEKA Elektronik, Lambrecht, D). In the BLM setup, a 1.5 mL Delrin cup with a 200-µm hole was fitted tightly into one of the two wells of a polymer bilayer chamber. The interior of the cup (*cis*) and the vacant well (*trans*) were filled with the electrolyte solution into which the two Ag/AgCl/1M KCl reference electrodes, connected to the amplifier’s head-stage, were immersed. Routinely, the *trans* electrode was voltage-clamped with respect to the *cis* electrode, which was connected to the ground pin of the amplifier headstage. BLM recordings were formed by painting *L*-α-phosphatidylchloline (azolectin) dissolved in hexane (50 mg.mL^−1^) over a cup aperture that had been treated earlier with a few µL of hexadecane/hexane (1∶100 v/v), and allowed to dry. For *Bps*Omp38 experiments the BLM had to display a capacitance of about 100 pF and give a stable, virtually leak-free current signal throughout minute-long recordings at constant potentials. After electrolyte (1 M KCl) was added to both sides of the BLM chamber, a stock solution of the purified *Bps*Omp38 (100 µg.mL^−1^ in 20 mM phosphate buffer, pH 7.5 and 0.2% (v/v) LDAO) was added into the *cis* chamber. *Bps*Omp38 insertions were induced when an external transmembrane potential of +200 mV or −200 mV was applied. Membrane current (*I*_m_) recordings were made at 25° with the membrane potential across the phospholipid bilayer kept at defined constant values between +/−25 mV and +/−150 mV. The acquired data were filtered with a 3-pole low-pass Bessel filter at 1 kHz and saved into the computer memory with a 1 ms (1 kHz) sampling interval. The membrane activity in terms of current flow was analyzed directly with PULSE acquisition software, or stored traces were handled with Microsoft Office Excel 2007 and GraphPad Prism *v.*5.0 (GraphPad Software Inc., San Diego, CA).

### Liposome Swelling Assays

The trimeric *Bps*Omp38 channel was reconstituted into liposomes as described previously [31], [32]. Soybean *L*-α-phosphatidylchloline (Sigma-Aldrich) (20 mg.mL^−1^, freshly prepared in chloroform) was used to form multi-lamellar liposomes. For proteoliposome preparation, 200 ng of *Bps*Omp38 was reconstituted into the liposomes by sonication, and 15% (w/v) dextran (*M*_r_ 40,000) was subsequently entrapped in the proteoliposomes. The isotonic solute concentration was determined by mixing different concentrations of *D*-raffinose (prepared in 20 mM HEPES buffer, pH 7.5) with the liposome suspension. The concentration of *D*-raffinose that produced no liposome swelling or shrinking was the ‘isotonic concentration’. This value was used for the adjustment of isotonic concentrations of other solutes. Twenty microliters of liposome or proteoliposome solution was diluted into 600 µL of the isotonic test solution in a 1-mL cuvette and mixed manually. The initial rate of swelling upon addition of the isotonic sugar solutions was monitored using a spectrophotometer with the wavelength set at 500 nm. The apparent absorbance change over the first 60 sec was used to estimate the swelling rate (s^−1^) using the equation: Φ = (1/A_i_)dA/dt, in which Φ is the swelling rate, A_i_ the initial absorbance, and dA/dt the rate of absorbance change during the first 60 s. The swelling rate for each sugar was normalized by setting the rate with the smallest sugar, *L*-arabinose (*M*_r_ 150), to 100%. The values shown are averages obtained from four to six determinations. Protein-free liposomes and proteoliposomes without sugars or antimicrobial agents were used as negative controls. The sugars tested were *D*-glucose (*M*_r_ 180), *D*-mannose (*M*_r_ 180), *D*-galactose (*M*_r_ 180), *N*-acetylglucosamine (GlcNAc, *M*_r_ 221), *D*-sucrose (*M*_r_ 342) and *D*-melezitose (*M*_r_ 522). Antimicrobial agents used in liposome swelling assays were meropenem (*M*_r_ 383), imipenen (*M*_r_ 299), doripenem (*M*_r_ 421), ceftoxitin (*M*_r_ 428), cefepime (*M*_r_ 481) and ceftazidime (*M*_r_ 637).

### Statistical analysis

The significance of antimicrobial susceptibility tests for *Bps* and for *E. coli* expressing *Bps*Omp38 variants against 14 antimicrobial agents was evaluated using ± log_2_ dilution analysis, following the previously described reports [33], [34], and one-way ANOVA, available in GraphPad Prism v. 5.0. The statistical significance of ion conductances and relative permeability rates of sugars and antimicrobial agents of the three *Bps*Omp38 variants was evaluated by one-way ANOVA.

## Results

### Antimicrobial Susceptibility of *Bps*

Levels of antibiotic resistance of our clinically-derived *Bps* strain were investigated using the twofold serial broth microdilution method. When compared with the most recently updated breakpoints recommended for *pseudomonas spp.* by EUCAST (Table 1), most antimicrobial agents tested against *Bps* had MIC (minimum inhibitory concentration) values higher than the breakpoint values for resistance. Ceftazidime and meropenem are the only two antibiotics with MIC values lower than the breakpoint values, indicting that this *Bps* strain is sensitive to these two antibiotics. Statistical analysis of the data presented in Table 1 was carried out as shown Table 2. Log_2_ dilution analysis for each antimicrobial agent in the absence and presence of PAβN gave MIC values that agreed completely within ±2 log_2_ dilution, with >92% agreement within ±1 log_2_ dilution. For most antibacterial agents, 67–100% of MIC values are the same (log_2_ dilution difference = 0). The only exception are imipenem and ciprofoxacin, where their essential agreement within the same concentration is 50%, and 58%, respectively. We additionally evaluated the significance of antimicrobial susceptibility of *Bps* using one-way ANOVA. The results of this analysis showed *p*-values >0.05 with all the tested antimicrobial agents, showing non-significant differences between MIC values determined in the presence and absence of PAβN.

**Table 1:** Antibiotic susceptibility of a clinically derived strain of *Burkholderia pseudomallei*.

Antibiotic	Breakpointfor resistance^a^		MIC value (µg.mL^−1^)	
	S ≤	R >	−PAβN	+PAβN
**Penicillin**				
Penicillin G	≤16	>16	1024^R^	1024^R^
Amoxycillin	–	–	256	256
**Cephalosporin**				
Cefoxitin	NA	NA	1024	1024
Ceftazidime	≤8	>8	2^S^	2^S^
Cefepime	≤8	>8	512^R^	512^R^
**Carbapenem**				
Meropenem	≤2	>8	4^S^	4^S^
Imipenem	≤4	>8	8	8
Doripenem	≤1	>2	>2048^R^	>2048^R^
**Fluoroquinolone**				
Norfloxacin	–	–	8	8
Ciprofoxacin	≤0.5	>1	4^R^	4^R^
**Quinolone carboxylic acid**				
Enrofloxacin	–	–	4	4
**Sulfonamide-trimethoprim**				
Cotrimoxazol	4	4	128^R^	128^R^
**Aminoglycoside**				
Kanamycin	–	–	16	16
Gentamicin	≤4	>4	32^R^	32^R^

The values presented are obtained from the experiments performed 4–6 times.

a Breakpoints defined for *Pseudomonas spp*. follow the EUCAST Clinical Breakpoint Table v. 4.0, valid from 2014-01-01 [26]. R, Resistant; S, Sensitive; NA, Not applicable;

-, No breakpoints. Susceptibility testing is not recommended. In order to simplify the EUCAST tables, the intermediate category is not listed.

**Table 2:** Statistical analysis of MIC values of *Bps* shown in Table 1 by one-way ANOVA and log_2_ dilution methods.

			Log_2_ dilution analysis							
Antibiotic	N^a^	ANOVA analysis	Distribution of MIC values within ± log_2_ dilution					%Agreement^c^		
		*p*-value	−2	−1	0	+1	+2	same	±1	±2
**Penicillin**										
Penicillin G	12	0.6733(NS)^b^	0	1	11	0	0	92	100(NS)	100(NS)
Amoxycillin	12	1.000(NS)	0	1	9	2	0	75	100(NS)	100(NS)
**Cephalosporin**										
Cefoxitin	12	0.7728(NS)	0	3	9	0	0	75	100(NS)	100(NS)
Ceftazidime	12	1.000(NS)	0	3	8	1	0	67	100(NS)	100(NS)
Cefepime	12	0.5862(NS)	0	2	9	1	0	75	100(NS)	100(NS)
**Carbapenem**										
Meropenem	12	0.7728(NS)	0	0	9	3	0	75	100(NS	100(NS
Imipenem	12	0.1736(NS)	1	2	6	3	0	50	92(NS)	100(NS)
Doripenem	12	0.7728(NS)	0	3	9	0	0	75	100(NS)	100(NS)
**Fluoroquinolone**										
Norfloxacin	12	1.000(NS)	1	2	8	1	0	67	92(NS)	100(NS)
Ciprofoxacin	12	0.1250(NS)	1	2	7	2	0	58	92(NS)	100(NS
**Quinolone carboxylic acid**										
Enrofloxacin	12	1.000(NS)	1	3	8	0	0	67	92(NS)	100(NS)
**Sulfonamide-trimethoprim**										
Cotrimoxazol	12	0.7728(NS)	0	0	9	3	0	75	100(NS)	100(NS)
**Aminoglycoside**										
Kanamycin	12	1.000(NS)	1	3	8	0	0	67	92(NS)	100(NS)
Gentamicin	12	0.7500(NS)	1	1	9	1	0	75	92(NS)	100(NS)

a N is the total number of samples used for both analyses. Two equal-sized sampling groups (each group, n = 6) were used, in the absence and presence of PAβN.

b NS represents non-significant difference between the two studied groups at *p*<0.05.

c NS represents non-significant difference between the two studied groups at essential agreement ≥ 85% [37].

### Recombinant Expression, Purification and Protein Identification

**Figure 1:**
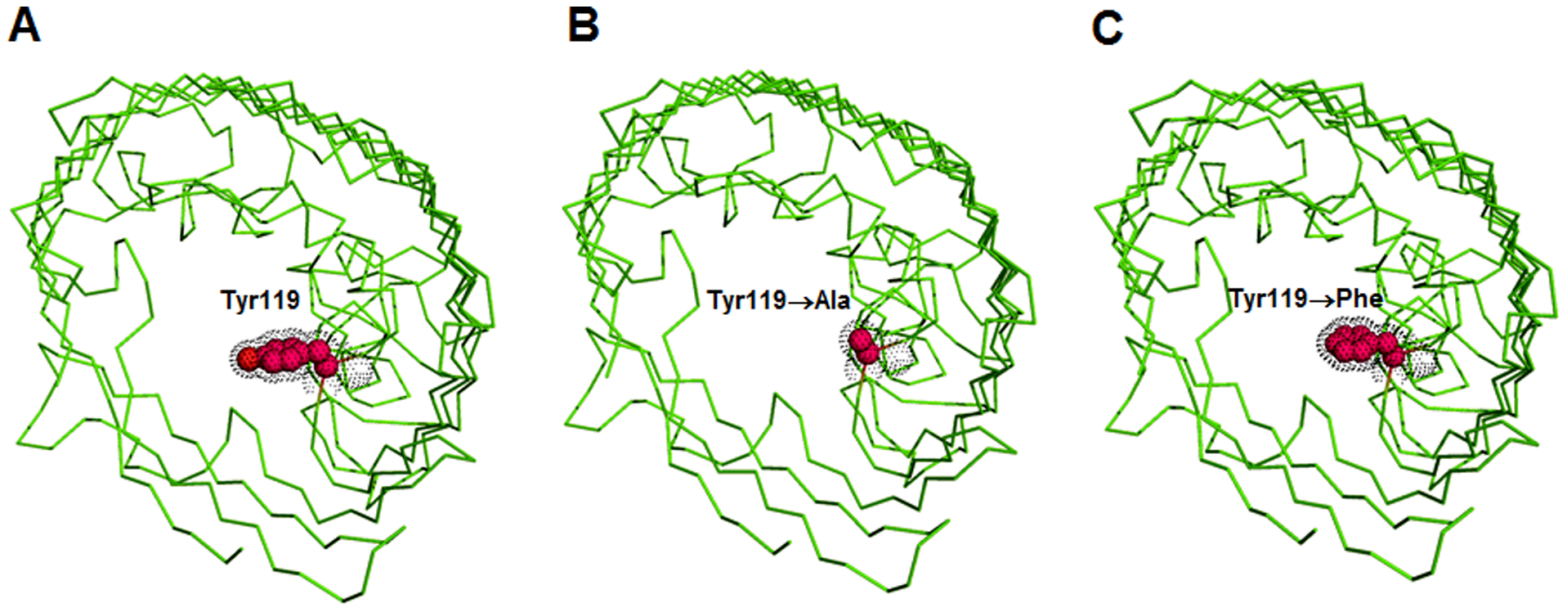
A ribbon representation of the homology model of *Bps*Omp38. The modeled structure of *Bps*Omp38WT (A) shows the key residue Y119 (red) protruding into the channel lumen (top view). This residue was mutated to Ala or Phe, generating two single mutants, *Bps*Omp38Y119A (B) and *Bps*Omp38Y119F (C).

In this study, we improved the *Bps*Omp38 expression system by re-cloning the gene encoding *Bps*Omp38, including the endogenous signal sequence to allow the recombinant protein to insert into the *E. coli* cell wall. The protein was then purified from the membrane fraction of the disrupted cells. We also carried out site-directed mutagenesis to investigate the importance of Tyr119 in regulating channel permeability. In the modelled 3D-structure, this residue is part of a short right-handed α-helix (Tyr119→Leu126) that precedes the longest loop 3 (L3) and its side chain is situated in the lumen of *Bp*sOmp38. Tyr119 may therefore be involved in controlling the passage of hydrophilic molecules through the *Bps*Omp38 pore. Figure 1 is a ribbon model of *Bps*Omp38WT, showing the sidechain of Tyr119 protruding into the centre of the pore (Fig. 1A). Figures 1B and 1C show the same view of the *Bps*Omp38WT pore, with Y119 virtually mutated to alanine and phenylalanine, respectively. Amino acid substitution of *Bps*Omp38 generated two single mutants, *Bps*Omp38Y119A and *Bps*Omp38Y119F, which differed in the properties of the side-chains at position 119, alanine being small and non-polar, whereas phenylalanine is aromatic and hydrophobic.

**Figure 2:**
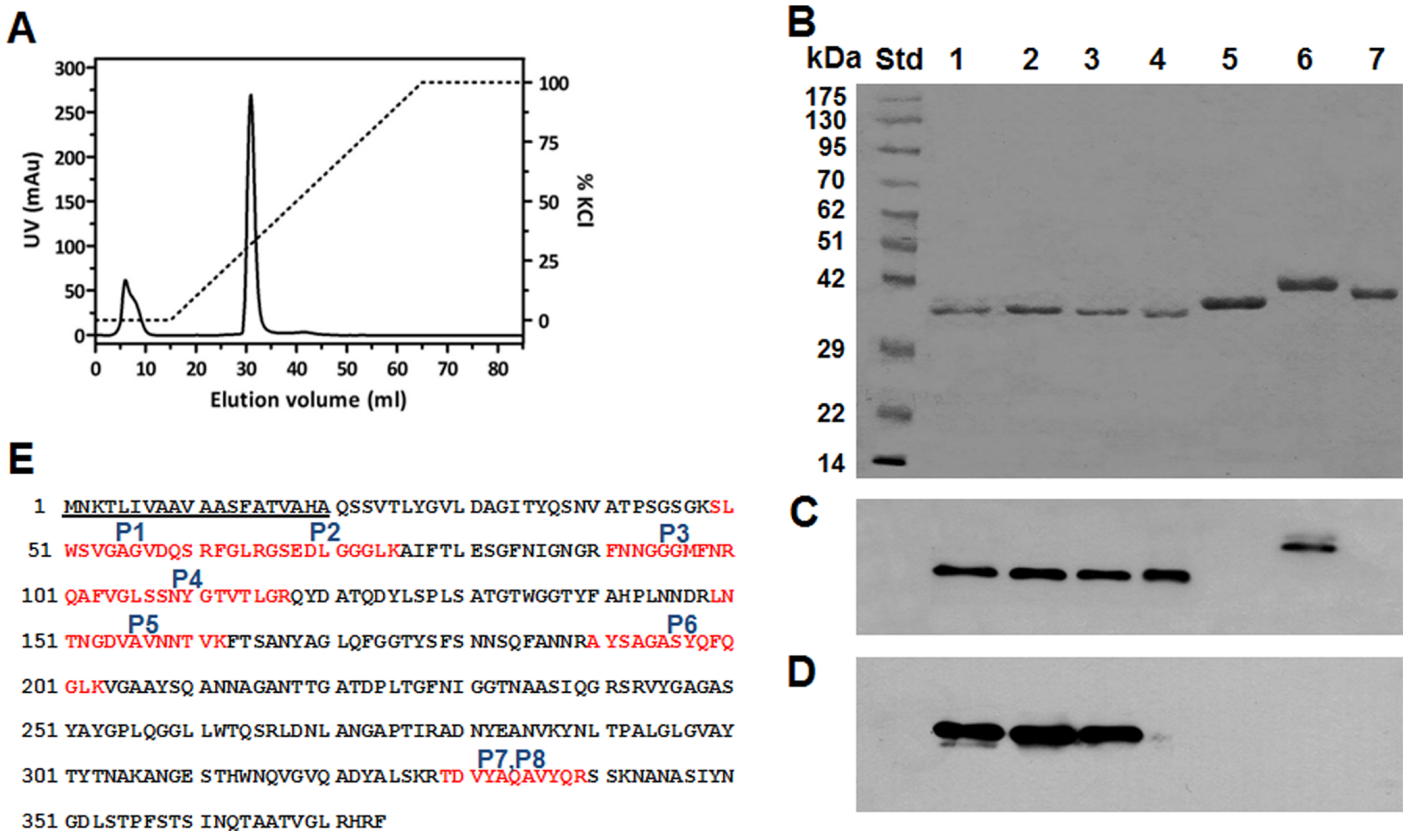
Purification and immunodetection of the *Bps*Omp38 variants, expressed in *E. coli*. (A) Chromatographic profile of *Bps*Omp38 purification with a Hitrap SP HP column, connected to an ÄKTA Prime plus FPLC system. The protein was eluted with a linear gradient of 0–1 M KCl, (B) SDS/PAGE followed by Coomassie Blue staining, (C) immunoblot analysis of the same samples as in panel B, using polyclonal anti-*Bps*Omp38 serum, (D) immunoblot analysis using anti-His_6_ monoclonal antibody, and (E) identification of tryptic digests of the expressed proteins by nanoLC/ESI MS (First BASE Laboratories, Malaysia). Eight peptides, designated P1–P8 and shown in red, were unambiguously identified by MASCOT as identical to internal sequences of *Bps*Omp38. The 20-aminoacid signal peptide is underlined. Lanes: Std, standard proteins; 1, *Bps*Omp38WT; 2, *Bps*Omp38Y119A; 3, *Bps*Omp38 mY119F; 4, refolded *Bps*Omp38; 5, *E. coli* OmpF; 6, *E. coli* OmpN; 7, and *V. harveyi* ChiP.

The recombinant *Bp*sOmp38 generated from the new construct was expressed as the (His)_6_-tagged polypeptide, and accumulated in the cell wall of the *E. coli* host cells. The peptidoglycan fraction was extracted with the detergent octyl-POE and the soluble extract, containing *Bps*Omp38, subjected to affinity chromatography, and then to ion exchange chromatography as described in *Materials and Methods*. Bound *Bps*Omp38 was eluted from the Hitrap SP HP ion exchange column. with an applied gradient of 0–1 M KCl, and Fig. 2A shows the elution profile of the recombinant *Bps*Omp38WT. Figure 2B shows a Coommassie blue-stained SDS-polyacrylamide gel, in which purified *Bps*Omp38 variants migrated as single bands slightly below 42 kDa. Figure 2C shows an immunoblot of the corresponding protein bands, detected with anti-*Bps*Omp38 polyclonal antiserum. Only *Bps*Omp38WT (lane 1), *Bps*Omp38Y119A (lane 2), *Bps*Omp38Y119F (lane 3) and refolded *Bps*Omp38 from our previous expression and purification protocol (lane 4) reacted strongly with the *Bps*Omp38 antiserum. Note that the *Bps*Omp38 antiserum also cross-reacted with a protein band identified as *E. coli* OmpN (lane 6), but not with *E. coli* OmpF (lane 5) or *Vibrio harveyi* chitoporin or ChiP (lane 7). When an immunoblot identical to that in Fig. 2B was probed with anti-(His)_6_ monoclonal antibody (Fig. 2D), only the protein bands in the first three lanes were labeled, while the other proteins, which lacked (His)_6_ tags, failed to react the with the antibody. These results confirmed that the porins expressed by the Omp-deficient *E. coli* host are *Bps*Omp38. To further verify this, tryptic peptides were generated by an in-gel digestion method and analyzed by mass spectrometry (First BASE Laboratories Sdn Bhd, Selangor Darul Ehsan, Malaysia). A MASCOT database search identified 11 peptides (three of which were redundant) that were identical to the internal sequences of an outer membrane porin from *Burkholderia pseudomallei* type strain 1655 (gene id. BURPS1655_I0506) (Table S1). These internal peptide sequences, labelled P1–P8, are shown in Fig. 2E and provide 25% coverage of the putative *Bps*Omp38 sequence identified previously [23].

### Effects of Tyr119 Mutations on Antimicrobial Susceptibility of the *E. coli* Expressing *Bps*Omp38

MIC values of the *E. coli* (Omp8) Rosetta strain expressing *Bps*Omp38WT and of the *E. coli* strain harboring an empty pET23d(+) vector (control *E. coli*) were compared. Table 3 shows that *E. coli* harboring pET23d(+) with and without *BpsOmp38* DNA insert had very high MIC values for penicillin G and amoxycillin (>2,048 µg.mL^−1^). However, when clavulanic acid, a known suicide inhibitor of β-lactamases (http://www.drugbank.ca/drugs/DB00766) was included in the culture medium, their MIC values were dramatically reduced, to 32 µg.mL^−1^ (Table 3, values in brackets). Apart from the penicillin class, for most antimicrobial agents the MIC values against *E. coli* expressing *Bps*Omp38 were one dilution higher than MIC values against control *E. coli*. Comparing the *E. coli* expressing three *Bps*Omp38 variants, MIC values for the *Bps*Omp38Y119A mutant were significantly lower than the WT values, particularly for the cephalosporins, (ceftazidime and cefoxitin), and the carbapenems (meropenem and imipenem). For *E. coli* expressing the *Bps*Omp38Y119F mutant, MIC values were significantly decreased for ceftazidime and gentamicin, while an increased MIC value was seen only with doripenem. For the other agents, MIC values for *Bps*Omp38Y119F equalled the WT values.

**Table 3:** Antimicrobial susceptibility of Omp-deficient *E. coli*, expressing *Bps*Omp38 variants.

Antibiotic		‘MIC value (µg.mL^−1^)				%Agreement within ±1 log_2_ dilution		
	N	Control	WT	Y119A	Y119F	*Control vs. WT*	*WT vs. Y119A*	*WT vs. Y119F*
**Penicillin**								
Penicillin G	10	>2048(16)^a^	>2048(32)*	>2048(16)**	>2048(16)**	100	80**	80**
Amoxycillin	10	>2048(32)	>2048(32)	>2048(32)	>2048(32)	100	90	100
**Cephalosporin**								
Ceftazidime	10	0.5	1*	0.5**	0.5**	80*	80**	80**
Cefoxitin	10	2	4**	2**	4	90	100	100
Cefepime	10	0.125	0.125	0.25	0.125	100	100	100
**Cabepenem**								
Meropenem	10	0.25	0.25	0.125**	0.25	100	100	100
Imipenem	10	2	4*	2**	4	90	100	100
Doripenem	10	128	256*	256	512**	100	100	90
**Fluoroquinolone**								
Norfloxacin	10	0.0625	0.125	0.125	0.125	100	100	100
Ciprofoxacin	10	≤0.03125	≤0.03125	≤0.03125	≤0.03125	100	100	100
**Quinolone carboxylic acid**								
Enrofloxacin	10	≤0.03125	≤0.03125	≤0.03125	≤0.03125	100	100	100
**Sulfonamide-trimethoprim**								
Cotrimoxazol	10	1	2	2	1	100	100	100
**Aminoglycoside**								
Kanamycin	10	256	512	512	256	100	100	100
Gentamicin	10	0.25	2**	1	0.5**	50**	80**	70**

Two statistical methods (ANOVA and log_2_ dilution) were used to evaluate the significance of their MIC values. Difference between the two studied groups is statically significant with P<0.05 using ANOVA analysis or with ≥85% essential agreement using log_2_ dilution analysis.

a Value in brackets is the MIC value when grown in the presence of antibiotic and clavulanic acid, a β-lactamase inhibitor.

*Significantly different MIC values for *E. coli* expressing *Bps*Omp38 WT and control *E. coli*.

**Significantly different MIC values for *E. coli* expressing *Bps*Omp38 WT and *E. coli* expressing *Bps*Omp38Y119A or *Bps*Omp38Y119F.

### Effects of Tyr119mutations on the Pore Conductance of the *Bps*Omp38 Channel

**Figure 3:**
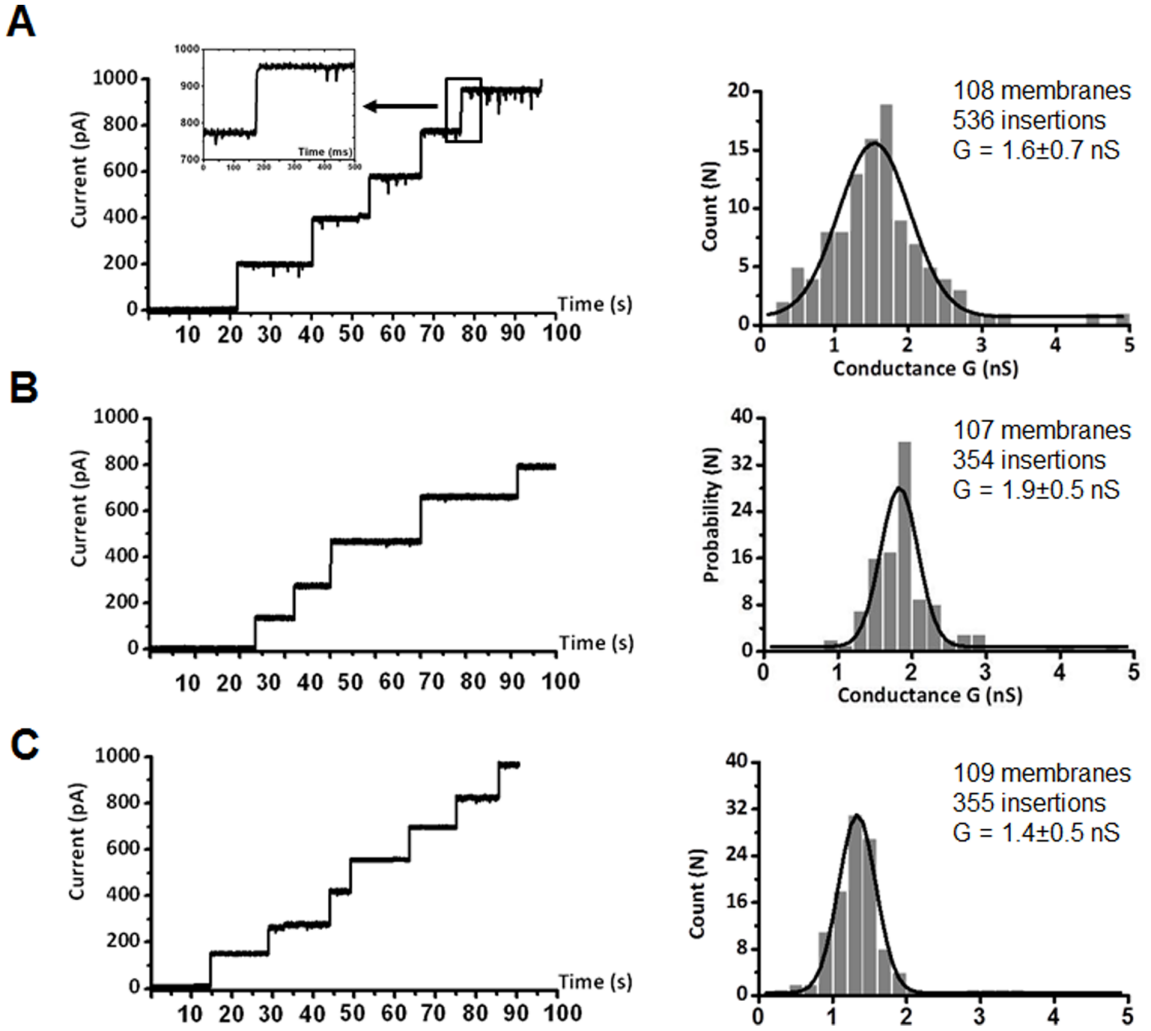
Ion current recordings obtained by the black lipid membrane (BLM) reconstitution technique. BLM measurements of successive insertions of *Bps*Omp38 trimers at a transmembrane potential of +100 mV. Lipid bilayers were formed across a 200-µM aperture by the ‘painting’ technique using 50 mg.mL^−1^ azolectin in *n*-hexane and bathed on either side in 1 M KCl. *Bps*Omp38 (1 µg.mL^−1^) was added on the *cis* side. (A) *Bps*Omp38WT, (B) *Bps*Omp38Y119A, and (C) *Bps*Omp38Y119F. Left panels are ion current traces acquired for 100 s. Fast insertion of one *Bps*Omp38WT channel, occurring within the millisecond time-resolution, was captured and shown as an inset. The traces represent multiple insertions of *Bps*Omp38 variants produced by applied membrane potentials of +100 mV. Right panels are histograms from the corresponding traces, giving the probability of a pore conductance (G) averaged over several hundred inserting channels as indicated. The black line represents a single Gaussian fit.

**Figure 4:**
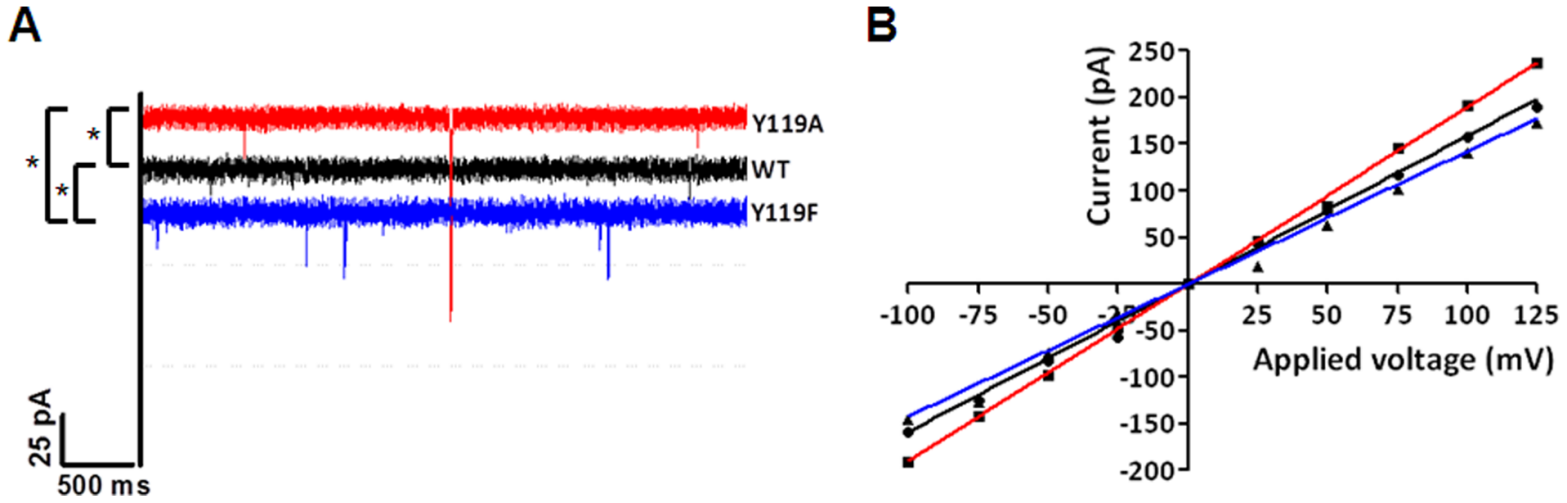
Single channel recordings of *Bps*Omp38 porin in artificial lipid membranes. Typical ion current traces of single *Bps*Omp38 variant channels in a fully open state at a transmembrane potential of +100 mM, (B) Current-voltage relationship of *Bps*Omp38WT in comparison with its two mutants. The current through a single open *Bps*Omp38 channel was monitored in 1 M KCl, following discrete changes in the voltage across the phospholipid membrane, from −100 to +125 mV. The slopes of a linear fit yielded the single channel conductances of individual *Bps*Omp38 channels. Data points for *Bps*Omp38WT, *Bps*OmpY119A and *Bps*OmpY119F are plotted as circles, squares and triangles, respectively. Differences in the three data sets were evaluated using one-way ANOVA. Statistically significant difference (P<0.05) is shown with an asterisk (*).

We further investigated the channel-forming properties of recombinant *Bps*Omp38 using the black lipid membrane (BLM) reconstitution technique. The purified protein was reconstituted into a freshly-formed phospholipid (azolectin) bilayer following addition on the *cis* (ground) side, the two sides of the chamber being filled with equal volumes of 1M KCl/20 mM phosphate buffer, pH 7.5. Insertions of *Bps*Omp38 channel were induced by applying a potential of +/−200 mV across the 200-µm diameter aperture. Employing the solvent-containing (so-called ‘painting’) BLM technique, multiple channels of the wild-type *Bps*Omp38 were frequently inserted (Fig. 3A, left panel) in a stepwise fashion until the ionic current reached 1 nA, beyond which point current signals could not be acquired, through limitations of the Dagan amplifier of the BLM setup. Note that the number of individual channels that could insert into the membrane depended on the potential applied. Figure 3A (right panel) is a histographic analysis, showing the statistical distribution of pore conductance averaged over 108 membranes and 535 channel insertions at +100 mV. For the wild-type channel (*Bps*Omp38WT), the average conductance was estimated to be 1.6±0.7 nS. Figure 3B (left panel) shows a typical insertion profile of the *Bps*Omp38Y119A mutant. Substitution of Tyr119 with alanine increased the ionic conductance of the *Bps*Omp38 channel to 1.9±0.5 nS (Fig. 3B, right panel), whereas substitution with phenylalanine (*Bps*Omp38Y119F mutant, Fig. 3C, left panel) decreased it to 1.4±0.5 nS (Fig. 3C, right panel). Although the Dagan BLM set up was not convenient for single channel measurement, insertion of a single *Bps*Omp38 molecule into the lipid bilayer could be achieved when very dilute protein (<20 ng.mL^−1^) was added into the *cis* chamber, allowing data acquisition from a single channel. Figure 4A shows the insertion of single *Bps*Omp38WT, *Bps*Omp38Y119A and *Bps*Omp38Y119F channels. The results confirmed that the conductance of the WT channel was lower than that of the Ala mutant, but higher than that of the Phe mutant. Ionic currents were also recorded at discrete voltages from +/−25 to +/−125 mV, and the resultant plots of ion current (I) *vs*. transmembrane potential (V) were found to be linear over the range –100 to +125 mV (Fig. 4B). The slopes of the I–V plots yielded single-channel conductances of 1.6±0.03 nS for *Bps*Omp38WT, 1.9±0.02 nS for *Bps*Omp38Y119A and 1.4±0.05 nS for *Bps*Omp38Y119F. These values corresponded well with the average conductances estimated from Gaussian fits of the distribution histograms obtained from multiple insertions, as shown in Fig. 3A–C.

### Effects of Tyr119 Mutation of Sugar and Antibiotic Permeabilities of *Bps*Omp38

**Figure 5:**
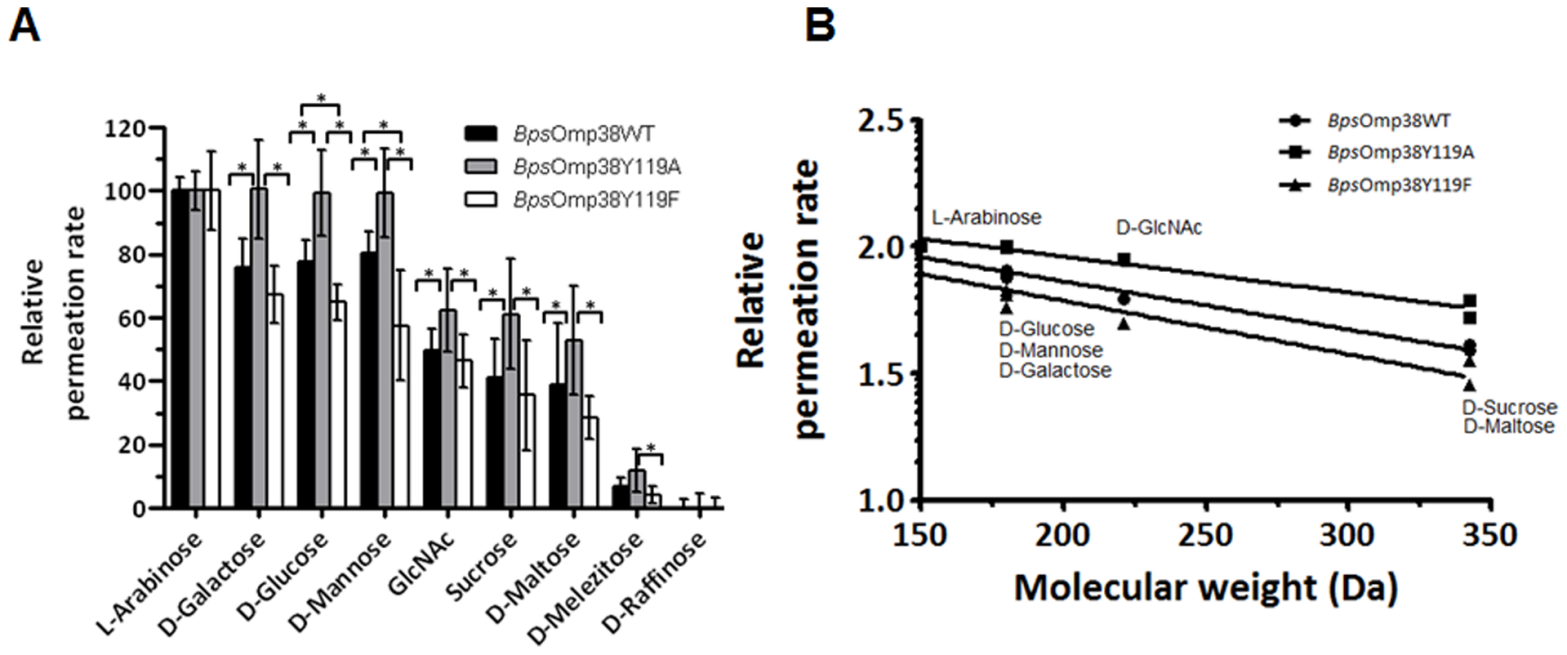
Swelling of *Bps*Omp38-containing proteoliposomes, induced by neutral sugars. For each preparation, multilamellar liposomes were reconstituted with 200*Bps*Omp38. The isotonic concentration was defined as the concentration of *D*-raffinose that caused no change in the absorbance at 500 nm of the proteoliposome suspension, over a period of 60 s. (A) Permeation of different types of sugars through *Bps*Omp38 reconstituted liposomes. Each swelling rate was normalized to the rate of swelling in arabinose, which was set to 100%. (B) Semilogarithmic plot of relative permeation rates of sugars through the proteoliposomes reconstituted with *Bps*Omp38WT and other mutants. The logarithm of the permeation rate is plotted against the molecular weight of the sugar. Differences in the three data sets were evaluated using one-way ANOVA. Statistically significant differences (P<0.05) are marked with an asterisk (*). Values are as means ± SD, obtained from 4–6 independent sets of experiments.

We further demonstrated involvement of the Tyr119 residue in sugar permeability (Fig. 5). The liposome swelling assay showed that the rate of swelling of *Bsp*Omp38-reconstituted liposomes, a measure of the rate of sugar permeation, decreased relative to the rate with the smallest sugar (*L*-arabinose) as the molecular size of sugar increased (Fig. 5A). The permeation rates of the selected neutral sugars were in the order: *L*-arabinose (*M*_r_ 150)>*D*-galactose ≅ *D*-glucose ≅ *D*-mannose (*M*_r_ 180)>*D*-GlcNAc (*M*_r_ 221)>*D*-sucrose (*M*_r_ 342). Little permeation of *D*-melezitose (*M*_r_ 522) or *D*-raffinose (*M*_r_ 504) was observed since the size of these sugars was close to the exclusion limit of *Bps*Omp38. Figure 5B shows that the logarithm of the permeation rate was linearly related to the molecular weight of the sugars. Figures 5A and B show that the rates with *Bps*OmpY119A were significantly higher, while the rates with *Bps*OmpY119F were lower than those with the wild-type.

**Figure 6:**
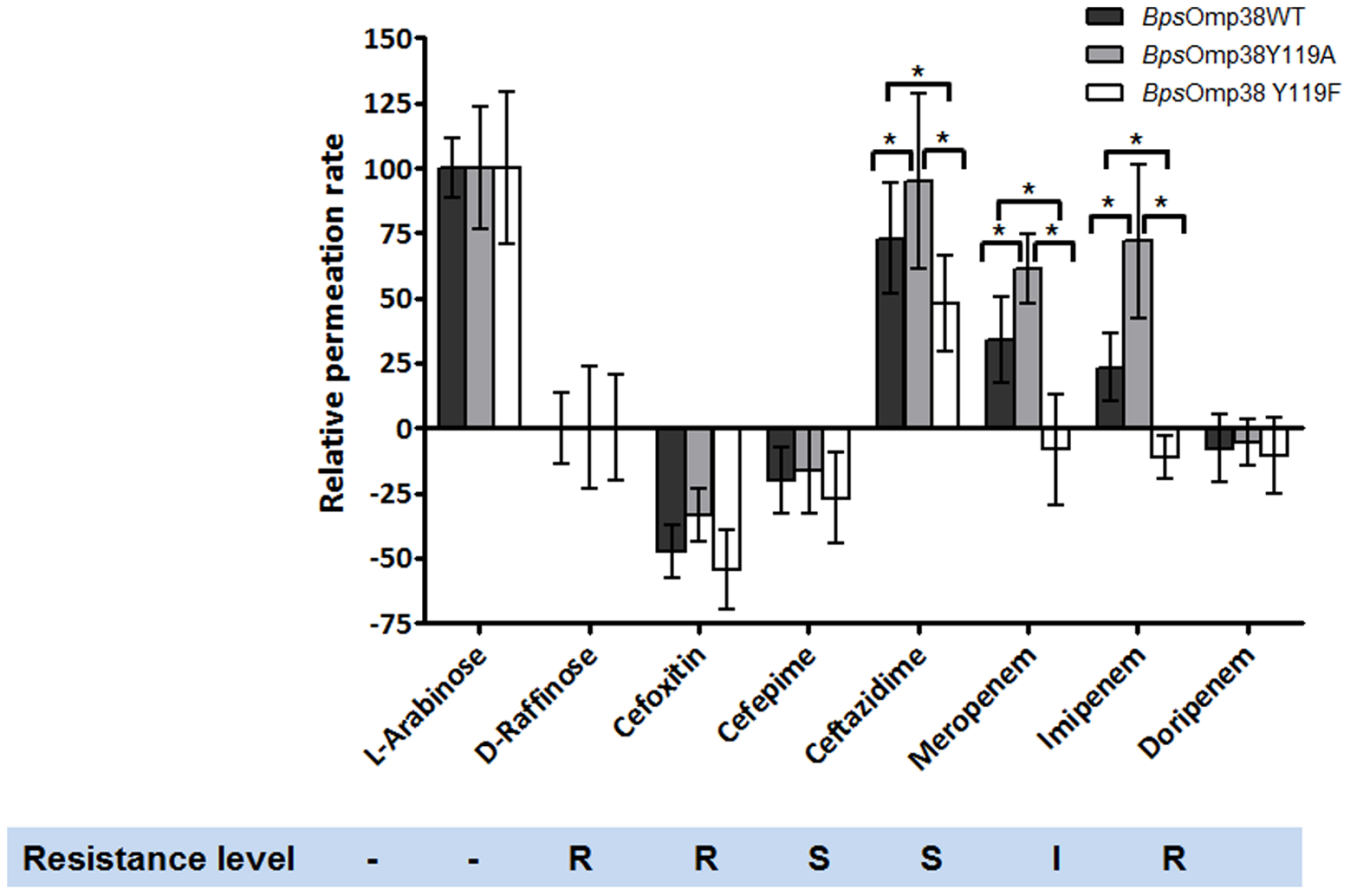
Swelling of *Bps*Omp38 proteoliposomes induced by cephalosporins and carbapenems. The proteoliposomes were prepared at pH–6 independent experiments. Levels of *Bps* resistance were determined by a broth micro dilution assay as presented in Table 1. R represents resistant; S, sensitive; and I, intermediate. Differences in the three data sets were evaluated using one-way ANOVA. Statistically significant differences (P<0.05) are marked with an asterisk (*). Values are as means ± SD, obtained from 4–6 independent sets of experiments.

Since expression of *Bps*Omp38 affected the susceptibility of *E. coli* to cephalosporins and carbapenems, we investigated the permeability to these antibiotics of the three *Bps*Omp38 variants. In the cephalosporin class, we compared the permeation rates of ceftazidime, to which *Bps* is sensitive, with those of cefoxitin and cefepime, to which *Bps* is resistant. Similarly in the carbapenem class, we compared the penetration rates of imipenem and meropenem, to which *Bps* is sensitive, with that of doripenem, to which it is resistant. The liposome swelling assays were performed at pH 7.5, to ensure that permeation occurred under physiological conditions; at this pH the net charge on the carbapenems and one of the cephalosporins (cefepime) is 0, while ceftazidime and cefoxitin have a net charge of −1 [35]. Figure 6 shows a relatively high rate of permeation of ceftazidime through *Bps*Omp38WT, with somewhat lower rates for meropenem and imipenem. On the other hand, the apparent permeation rates of cefoxitin, cefepime and doripenem are negative, indicating shrinkage of the liposomes, and therefore that the antibiotics were impermeant. The permeation of cephalosporin and carbapenem antibiotics through *Bps*Omp38 mutant channels was also investigated. The *Bps*Omp38Y119A mutant showed higher permeability toward the tested antimicrobial agents than *Bps*Omp38WT, while the permeability was dramatically decreased in the *Bps*Omp38Y119F mutant.

## Discussion

Antimicrobial resistance levels of our clinically-derived *Bps* strain were evaluated. As shown in Table 1, most MIC values were above the breakpoint values, indicating that this *Bps* strain is intrinsically resistant to most antimicrobial agents, and sensitive only to ceftazidime and meropenem. When compared with the 65 *Bps* isolates reported previously by Thaibault et al. [36], our *Bps* strain had greater resistance to amoxicillin, cefoxitin, imipenem, and ciprofoxacin, but lower resistance to norfloxacin. Although amoxicillin, imipenem, and co-trimoxazole are commonly prescribed for melioidosis treatment [9]–[13], our data clearly indicated that such antimicrobial agents would not be applicable to this *Bps* strain. This *Bps* exhibited particularly high resistance to three antimicrobial agents, penicillin G, cefoxitin and doripenem, with MIC values of 1,024 to >2,048 µg.mL^−1^. Statistical analysis shows essential agreement of 100% within ±2 log_2_ dilution and >92% within ±1 log_2_ dilution which is larger than a threshold for the log_2_ dilution analysis (85% essential agreement) [37], indicating that the differences of MIC values with and without PAβN present are insignificant. The correlation of the two-studied groups for all 14 antibiotics yielded *p*>0.05 in the one-way ANOVA analysis, further confirming insignificant differences in the MIC values with and without PAβN. Therefore, both statistical analyses clearly suggested that the intrinsic resistance of *Bps* to the drugs tested is not mediated by RND-type multidrug efflux pumps.

We previously expressed recombinant *Bps*Omp38 in inclusion bodies that required unfolding/refolding treatment and often yielded only small (µg) quantities of the purified protein, which were insufficient for thorough functional characterization. Our new protocol allowed trimeric *Bps*Omp38 to be expressed and inserted in the cell wall of the *E. coli* host cells, from which it was purified to homogeneity. As shown in Fig. 2, the purified protein, which migrated as a band of 42 kDa on SDS-PAGE, was equivalent to *Bps*Omp38 isolated from the outer membrane of native *Bps* [22]. After purification, 1–2 mg of recombinant *Bps*Omp38 per litre of bacterial culture was obtained; this was 5–10 times higher than was obtained using the unfolding/refolding protocol [22]. Although in the *E. coli* BL21 (Omp8) Rosetta strain the genes encoding OmpF, OmpC, OmpA and LamB are disrupted [25], our previous study showed that this bacterium could still express a significant amount of endogenous OmpN, as a major contaminant [38]. Although the *OmpN* gene is quiescent [39], with deficiency of the usual porin expression OmpN seemed to be induced, probably as an adaptive response to the nutritional stress and the need for the maintenance of normal growth. However, *E. coli* harboring the heterologous gene used *Bps*Omp38 as a major conduit for molecular uptake instead. This is supported by the data in Table 3, which shows some changes in the antimicrobial susceptibility of *E. coli* expressing *Bps*Omp38 (column 2) as compared with the control *E. coli (*column 1).

*E. coli* harboring pET23d(+) with and without the *BpsOmp38* DNA insert had exceptionally high MIC values for β-lactam antimicrobial agents. The pET23d(+) vector contains the *β*-lactamase gene in the same orientation as the target gene, so the high resistance presumably resulted from the degradation of the penicillin antibiotics by *β*-lactamase. This was verified by the reduction of MIC values from >2,048 µg.mL^−1^ to 32 µg.mL^−1^ (Table 3, values in brackets) for both penicillin G and amoxycillin when clavulanic acid, a suicide inhibitor of *β*-lactamases, was included in the culture medium. These results suggested that *β*-lactamase conferred on *E. coli* a high level of resistance towards the *β*-lactam antibiotics. The MIC value for kanamycin of *E. coli* harboring both empty and recombinant plasmid was also high, since this pET-series vector carries the kanamycin resistance gene.

*In vivo* investigation in the exogenous *E. coli* system suggested that MIC values of all antimicrobial agents against *E. coli* expressing *Bps*Omp38WT were generally higher than in control cells, which were transfected with a vector containing no insert. These control cells continued to express OmpN to support growth and was presumably responsible for the observed susceptibility to antimicrobial agents, and over-expression of *Bps*Omp38 apparently caused suppression of OmpN expression. As already shown, increased MIC values in *E. coli* expressing *Bps*Omp38 suggest that recombinant *Bps*Omp38 is used for entry of antibiotic molecules into these cells, its lower permeability lowering the antimicrobial susceptibility of the *E. coli* strain. *Bps*Omp38 has poor permeability towards most antimicrobial agents, hence causing decreases in antimicrobial susceptibility in the *E. coli* expressing *Bps*Omp38 as shown in Table 3.

Log_2_ dilution analysis (Table 3, column Control & WT) suggests that the difference in MIC values of the two groups was significant (essential agreement of ≤ 85%) with ceftazidime and gentamicin. Since log_2_ dilution analysis gave insufficient statistical resolution, we further carried out ANOVA analysis using a non-parametric correlation (Spearman) test. One-way ANOVA suggests significant differences (P<0.05, values marked with an asterisk) with a broader range of antimicrobial agents, including penicillin G, ceftazidime, cefoxitin, imipenem, doripenem, co-trimoxazone, and gentamicin. In *E. coli* expressing *Bps*Omp38 Y119A, susceptibility towards certain cephalosporins and carbapenems was regained (Table 3). Again, log_2_ dilution analysis shows significant difference with penicillin G, ceftazidime and gentamicin. On the other hand, ANOVA analysis shows significance with additional antibiotics, including cefoxitin, meropenem and imipenem. The limited signicance of mean values with log_2_ dilution analysis may reflect inadequate sample sizes. For ANOVA, small but equal sample sizes are generally accepted to give reliable results. The decreasing MIC values with *E. coli* expressing *Bps*OmpY119A mutant agree well with the enlargement of the pore conductance (as seen in Fig. 4) as a result of the replacement of the bulky tyrosine side chain with a smaller one. In contrast, *E. coli* expressing the *Bps*Omp38Y118F mutant had decreased susceptibility to the same groups of antimicrobial agents. This may be associated, in part, with the reduced pore conductance due to the ‘greasy’ phenylalanine side chain at position 119.

The results obtained from our *in vivo* studies indicated that expression of exogeneous *Bps*Omp38 altered susceptibility of the Omp-deficinent *E.* coli host to antimicrobial agents of the cephalosporin and carbapenem classes. In analogous work, a recent study by Bajaj *et al*. [40] showed that OmpPst1 porin of *Providencia stuartii,* an opportunistic pathogen found in patients with hospital-acquired urinary tract and wound infections, developed low membrane permeability as a strategy of resistance against imipenem. Using high-resolution BLM reconstitution measurements, they demonstrated that imipenem blocked the OmpPst1 pore, leading to a progressive decrease in the pore conductance as the concentration of imipenem increased. The low permeability of OmpPst1 was further revealed *in vitro* by liposome swelling assays, which showed slow permeation through the OmpPst1 channel by imipenem.

Small, neutral sugars exhibited reduced rates of permeability through *Bps*Omp38, with increasing molecular size. The logarithm of the relative permeability rate is inversely proportional to the molecular weight of the sugar, which is characteristic of general diffusion porins, as previously described [23]. The swelling rates of the proteoliposomes in *D*-galactose, *D*-glucose, and *D*-mannose were identical since these sugars are diastereomers of equal size. The permeation rates of the sugars through the *Bps*Omp38Y119A mutant were measured and were found to be significantly greater than the rates of permeation through *Bps*Omp38WT; in contrast, the permeation rates of these sugars were lower in the *Bps*Omp38Y119F mutant.

**Figure 7:**
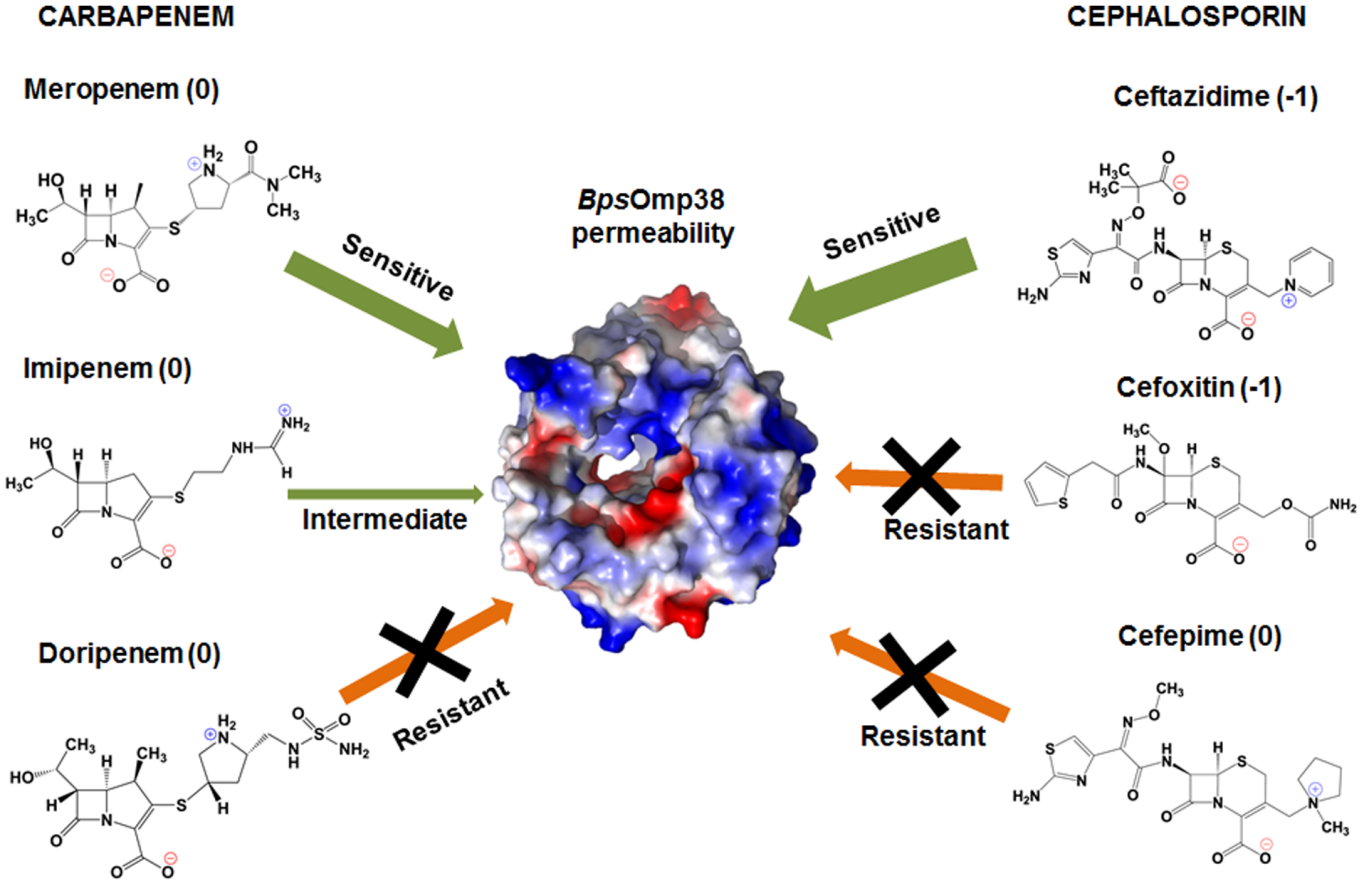
Summary of relative permeabilities of *Bps*Omp38 and the levels of *Bps* resistance to cephalosporin and carbapenem, together with the structures of the drugs. The value in brackets is charge state of each antibiotic at pH[35].

Unlike sugars, the relative permeability of antimicrobial agents through *Bps*Omp38 was not correlated with their molecular sizes, as shown in Fig. 6. Furthermore, liposome swelling rates in ceftazidime and cefoxitin were completely different, although these antimicrobial agents carry the same net charge; apparently the permeation of these two antibiotics depends on the molecular structure of the drugs, which affects their interaction with the channel. The catalytic role of antibiotic/porin interactions in facilitating drug permeation was first demonstrated for OmpF [41], with this facilitation effect further analyzed quantitatively [42]. Our results with of *Bps*Omp38 showed no permeability to cefoxitin, cefepime, or doripenem, while its high permeability to ceftazidime and moderate permeability to meropenem had a strong correlation with resistance of *Bps*, shown by the MIC values in Table 1. The relative permeation of *Bps*Omp38 mutants showed that substitution of the bulky chain of Tyr119 with the smaller one of alanine, minimizing steric hindrance, enhances the channel permeability, while substitution with the hydrophobic side chain of phenylalanine hindered the passage of the antibiotics. These results confirm the important role of Tyr119 in defining the membrane permeability of *Bps*. Figure 7 summarizes the data obtained from microbiological and biochemical assays, both of which suggest a correlation of the susceptibility of *Bps* to cephalosporin and carbapenem drugs with the permeability of the *Bps*Omp38 channel.

## Conclusion

Taken together the data obtained *in* *vitro* and *vivo* provide strong evidence that *Bps*Omp38 participates in resistance of *Bps* to the studied antimicrobial agents and that Tyr119, located prominently in the channel lumen, is an essential residue that takes part in drug-porin interactions.

## Supporting Information

Table S1 Identification of tryptic peptides by nano LC/ESIMS.
(DOC)
