# Supplementary material for: Porin Involvement in Cephalosporin and Carbapenem Resistance of Burkholderia pseudomallei
Source: PLoS One. 2014 May 1;9(5):e95918. doi: 10.1371/journal.pone.0095918 (PMC4006797; doi:10.1371/journal.pone.0095918)
Supplement: Table S1 — Identification of tryptic peptides by nano LC/ESIMS. (DOC) [file pone.0095918.s001.doc]

**SUPPLEMENTARY**

**Table S1.** Identification of tryptic peptides by nano LC/ESIMS.

After submission of the isotopic masses to a MASCOT database search, eight peptides were identified to be unambiguously compatible with the internal peptides of an outer membrane porin from *Burkholderia pseudomallei* 1655 (gene id. BURPS1655_I0506).

| **Peptide no*** | **Start** | **–** | **End** | **Observed** | **Charge** | ***M*_r_(expt)** | ***M*_r_(calc)** | **Peptide** |
| --- | --- | --- | --- | --- | --- | --- | --- | --- |
| 1 | 49 | – | 61 | 454.4741 | +3 | 1360.4003 | 1360.6735 | K.SLWSVGAGVDQSR.F |
| 1 | 49 | – | 61 | 1362.1779 | +1 | 1361.1706 | 1360.6735 | K.SLWSVGAGVDQSR.F |
| 2 | 62 | – | 75 | 469.4582 | +3 | 1405.3528 | 1404.7361 | R.FGLRGSEDLGGGLK.A |
| 3 | 91 | – | 100 | 565.2778 | +2 | 1128.5410 | 1128.4771 | R.FNNGGGMFNR.Q + Oxidation (M) |
| 4 | 101 | – | 117 | 590.5537 | +3 | 1768.6394 | 1768.9108 | R.QAFVGLSSNYGTVTLGR.Q |
| 5 | 149 | – | 162 | 730.2538 | +2 | 1458.4930 | 1457.7474 | R.LNTNGDVAVNNTVK.F |
| 5 | 149 | – | 162 | 1459.8120 | +1 | 1458.8047 | 1457.7474 | R.LNTNGDVAVNNTVK.F |
| 6 | 190 | – | 203 | 745.9739 | +2 | 1489.9333 | 1489.7201 | R.AYSAGASYQFQGLK.V |
| 7 | 328 | – | 339 | 735.4683 | +2 | 1468.9220 | 1468.7423 | K.RTDVYAQAVYQR.S |
| 7 | 328 | – | 339 | 735.5847 | +2 | 1469.1548 | 1468.7423 | K.RTDVYAQAVYQR.S |
| 7 | 328 | – | 339 | 490.9310 | +3 | 1469.7713 | 1468.7423 | K.RTDVYAQAVYQR.S |
| 8 | 329 | – | 339 | 657.4061 | +2 | 1312.7977 | 1312.6412 | R.TDVYAQAVYQR.S |

*Numbers of peptides identified by MS/MS are labeled. Peptides with identical sequences are assigned the same number.
